# Differences in Wall Shear Stress Between High-Risk and Low-Risk Plaques in Patients With Moderate Carotid Artery Stenosis: A 4D Flow MRI Study

**DOI:** 10.3389/fnins.2021.678358

**Published:** 2021-08-11

**Authors:** Guiling Zhang, Shun Zhang, Yuanyuan Qin, Jicheng Fang, Xiangyu Tang, Li Li, Yiran Zhou, Di Wu, Su Yan, Weiyin Vivian Liu, Wenzhen Zhu

**Affiliations:** ^1^Department of Radiology, Tongji Hospital, Tongji Medical College, Huazhong University of Science and Technology, Wuhan, China; ^2^Magnetic Resonance Research, General Electric Healthcare, Beijing, China

**Keywords:** plaque risk, 4D flow MRI, wall shear stress, high resolution vessel wall imaging, stroke

## Abstract

This study aimed to evaluate the difference in wall shear stress (WSS) (axial, circumferential, and 3D) between high-risk and low-risk plaques in patients with moderate carotid artery stenosis and to identify which time points and directions play the dominant roles in determining the risk associated with plaques. Forty carotid arteries in 30 patients were examined in this study. All patients underwent high-resolution vessel wall (HRVW) imaging, diffusion-weighted imaging (DWI), and 4D flow MRI; HRVW imaging and DWI were used to separate low- and high-risk plaque. Twenty-four high-risk plaques and 16 low-risk plaques were enrolled. An independent-sample *t*-test was used to compare WSS between low- and high-risk plaques in the whole cardiac cycle and at 20 different time points in the cardiac cycle. The study found that patients with high-risk plaques had higher WSS than those with low-risk plaques throughout the entire cardiac cycle (*p* < 0.05), but the changes varied at the 20 different time points. The number of non-significant differences (*p* > 0.05) was less in diastole than in systole across different time points. The axial WSS values were higher than the circumferential WSS values; the difference in axial WSS values between high- and low-risk plaques was more significant than the difference in circumferential WSS, whereas 3D WSS values best reflected the difference between high-risk and low-risk plaques because they showed significant differences at every time point. In conclusion, increased WSS, especially during the diastolic period and in the axial direction, may be a signal of a high-risk plaque and may cause cerebrovascular events in patients with moderate carotid artery stenosis. Additionally, WSS can provide hemodynamic information and help clinicians make more appropriate decisions for patients with plaques.

## Introduction

High-risk plaques that easily rupture and cause thrombosis or embolism are the predominant cause of cerebrovascular events (Falk et al., 2013). Conservative treatments such as pharmacological interventions can be used in patients with low-risk plaques, while proactive treatment such as stent placement or endovascular thrombectomy is usually utilized in those with high-risk plaques (Adamson et al., 2015). Plaques with a stenosis rate greater than 70% are independently associated with acute ischemic stroke and are defined as high-risk plaques, whereas patients with less than 30% stenosis are treated with medicine or prescribed for imaging follow-up (Bodle et al., 2013). However, for patients with moderate stenosis (a stenosis rate of 30–70%), treatments cannot be selected based on the stenosis alone; further examinations are needed to identify the high-risk plaques and take proactive treatments (Kernan et al., 2014; Bonati et al., 2015; Brott et al., 2016). Therefore, detecting high-risk plaques with moderate stenosis is of great importance.

High-risk plaques tend to be characterized by a large plaque volume, a necrotic lipid core, positive remodeling, peripheral neovascularization, a thin fibrous cap, microcalcification, intraplaque hemorrhage (IPH) and chronic inflammation (Adamson et al., 2015). Carotid endarterectomy provides the pathological tissue sections needed to identify between high- and low-risk plaques. However, this procedure is invasive; therefore, there is a pressing need for an accurate imaging method to identify high-risk and low-risk plaques in patients with moderate stenosis. Advanced imaging methods available to identify plaque risk are generally classified according to their basis: morphology or hemodynamics. A breakthrough has been made in morphological assessment with the development of high-resolution vessel wall (HRVW) imaging (Cai et al., 2005; Makowski et al., 2011; Qiao et al., 2014). HRVW can help identify stroke mechanisms, determine the degree and pathology of stenoses, and identify non-stenotic plaques and potentially high-risk plaque components (Bodle et al., 2013), but it still cannot completely distinguish high-risk from low-risk plaques by morphology alone, especially in the case of atypical plaques, due to limitations in resolution, an uncertain relationship with pathology, and morphological complexity and diversity. Therefore, the application of hemodynamics in combination with HRVW is useful and even essential in the determination of plaque risk.

Wall shear stress (WSS) is the most commonly reported risk indicator in hemodynamic research and is considered the most useful hemodynamic parameter for assessing plaques. It is generally accepted that low WSS may promote plaque formation by activating inflammatory processes (Matlung et al., 2012; Peiffer et al., 2013; Hung et al., 2015; Zhang et al., 2017). However, it remains controversial whether high WSS causes plaque rupture (Groen et al., 2008) or protects plaques from rupturing (Malek et al., 1999; Cheng et al., 2006). Further studies are needed to identify the relationship between WSS and plaque risk. 4D flow MRI is a novel method to non-invasively measure hemodynamic parameters *in vivo*. Compared to the traditional method, computational fluid dynamics (CFD), which relies on a fluid mechanics model based on idealized assumptions in a simulation to acquire hemodynamic information, 4D flow MRI directly measures blood velocity to compute hemodynamic values. The results of 4D flow MRI results are considered more realistic and reliable than those of CFD (Dyverfeldt et al., 2015). In addition, 4D flow MRI can dynamically and visually display hemodynamic changes across different cardiac cycles and can be used to explore the relationship between high- and low-risk plaques for different flow directions and plaque locations (Sotelo et al., 2018; Rizk et al., 2019).

HRVW imaging combined with the 4D flow MRI technique was used in our study to explore the differences in WSS between high- and low-risk plaques in patients with moderate stenosis. We aim to identify whether WSS is a predictor of high-risk plaques and which directions of blood flow and phases of the cardiac cycle play the most important roles in atherosclerosis. This study provides information on the hemodynamic aspects of plaque imaging and can help with further clinical treatments in atherosclerotic patients.

## Materials and Methods

### Subjects

**FIGURE 1:**
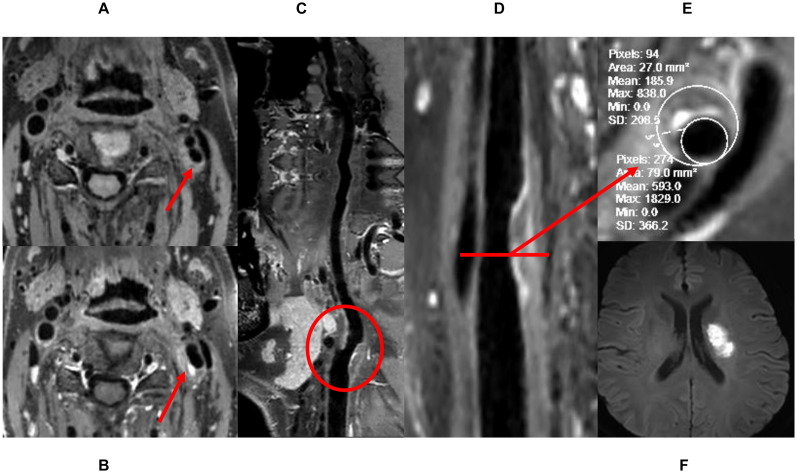
The definition of high-risk plaque. **(A)** Pre-enhanced HRVW 3D T1 image, inhomogeneous intensity was seen in the plaque. **(B)** Post-enhanced HRVW 3D T1 image. Hyperintensity was seen in the plaque. **(C,D)** Reconstruction map of post-enhanced HRVW 3D T1 image. The whole vessel and a plaque shown in red circle were better displayed. **(E)** Section image perpendicular to the lumen in the plaque lesion. Stenosis rate was determined in this section. **(F)** DWI. Hyperintensity was seen in left basal ganglia, indicating cerebrovascular events occurred.

**FIGURE 2:**
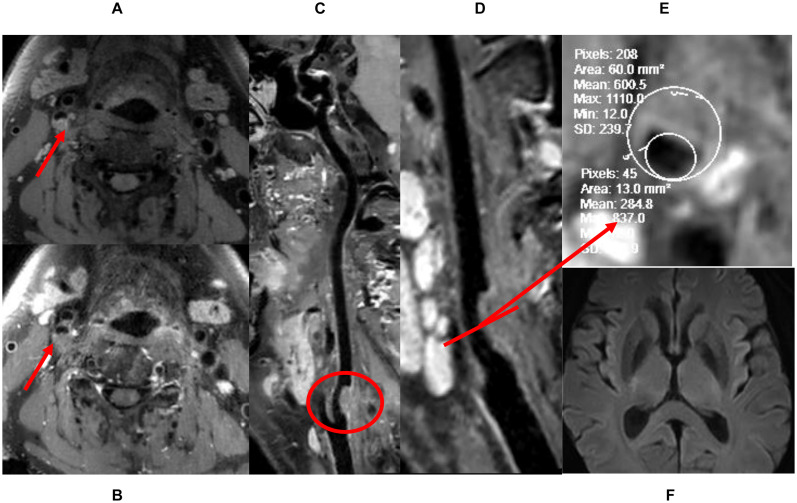
The definition of low-risk plaque. **(A)** Pre-enhanced HRVW 3D T1 image. Intensity is homogeneous was seen in the plaque. **(B)** Post-enhanced HRVW 3D T1 image. No abnormal intensity was seen in the plaque. **(C**,**D)** Reconstruction map of post-enhanced HRVW 3D T1 image. Better display of the whole vessel and the plaque shown in red circle. **(E)** Section image, perpendicular to the lumen located in the plaque lesion. The stenosis rate can be determined in this section. **(F)** DWI. No abnormal hyperintensity was found in the whole brain, indicating no cerebrovascular events occurred.

We recruited 34 patients with carotid plaques from January 2019 to June 2020. The stenosis rate of all plaques was initially assessed to be 30–70%, three patients with low-quality imaging and one patient whose actual stenosis rate was greater than 70% were excluded from the study. Ultimately, 40 carotid arteries of 30 patients were included: 10 patients had bilateral carotid plaques, and the 20 remaining patients had one healthy carotid artery each. Twenty-four had high-risk plaques, and 16 had low-risk plaques. High-risk plaques were defined as follows: (1) HRVW imaging: showed potentially high-risk plaque characteristics, including heterogeneous signal, a thin fibrous cap, IPH, a lipid core, or obvious partially enhanced signal. The enhancement was quantified as follows: [signal intensity of plaque (post-contrast)/signal intensity of gray matter (post-contrast)]/[signal intensity of plaque (pre-contrast)/signal intensity of gray matter (pre-contrast)]; when this value was greater than 1, the plaque was enhanced. (2) Cerebrovascular events had occurred, with definite hyperintense lesions identified in the ipsilateral brain parenchyma on DWI (Figure 1). Low-risk plaques were defined as follows: (1) HRVW imaging: did not show potentially high-risk plaque components and did not show enhancement. (2) No obvious lesion was identified in the ipsilateral brain parenchyma on DWI (Figure 2; Adamson et al., 2015; Zhang R. Y. et al., 2020).

### Examination Protocol

All patients underwent 4D flow MRI, HRVW 3D T1-weighted imaging (T1WI), and DWI. The 4D flow MRI scans were conducted using a 3.0-T MRI scanner (GE Medical Systems, Discovery MR750, Waukesha, WI, United States) with an eight-channel head–neck coil. The 4D flow MRI data were acquired using a volumetric, time-resolved phase-contrast method. The scanning parameters were as follows: TR = 4.1 ms, TE = 2.1 ms, flip angle = 8°, FOV = 240 × 240 mm^2^, matrix size = 128 × 128, NEX = 1 and receiver bandwidth = ± 62.5 kHz. Velocity encoding (VENC) was set at 100 cm/s to prevent aliasing artifacts. Twenty frames were reconstructed through view sharing. The total scan time of the 4D flow was approximately 4–6 min depending on the heart rate of each subject (Zhang G. et al., 2020). DWI and HRVW imaging were conducted immediately after the 4D flow MRI. A 32-channel head coil combined with a soft coil attached to the neck with only a slight space between neck and neck coil was used at a 3T MR scanner (UMR780, United Imaging Healthcare, Shanghai, China). The DWI parameters were as follows: TR = 4,049 ms, TE = 48 ms, thickness = 5 mm, flip angle = 90°, FOV = 230 × 220 mm^2^, B values = 0 and 1,000 s/mm^2^, voxel size = 1.60^∗^1.44^∗^5.00 mm, and scan time 1min54s. HRVW imaging was performed with 3D-TIWI matrix before and after an injectable gadolinium-based contrast agent, gadobenate dimeglumine injection (Bracco Sine Pharmaceutical Corp., Ltd.; Shanghai, China), was administered intravenously (0.1 mmol/kg of body weight); 5 min after the contrast agent was administered, we conducted post-enhancement HRVW. The parameters were as follows: TR = 750 ms, TE = 23.7 ms, FOV = 220 mm × 180 mm, slice thickness = 0.66 mm, voxel size = 0.65 × 0.65 × 0.66 mm, and scan time = 7 min 13 s.

### Data Analysis

**FIGURE 3:**
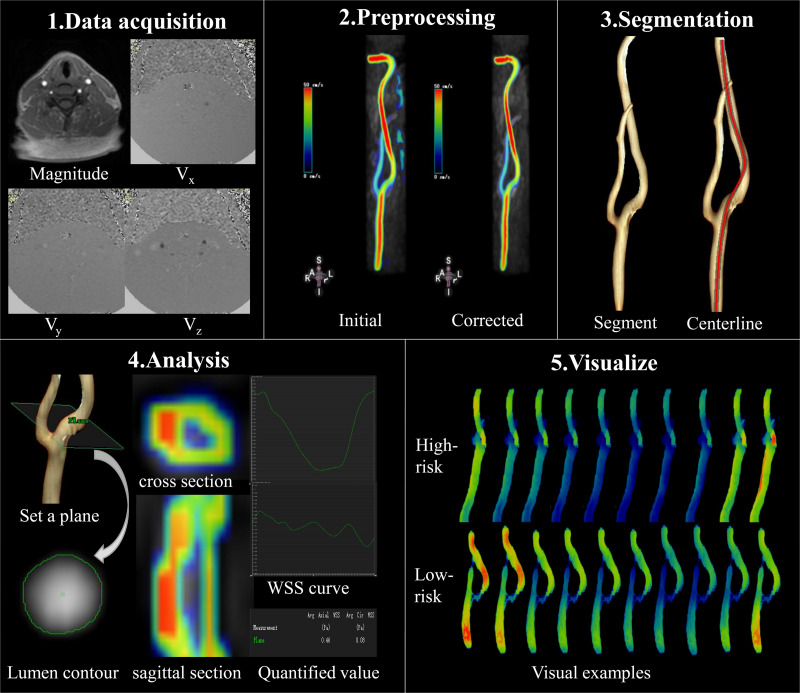
Data acquisition and analysis workflow for 4D flow MRI. **(1)** Data acquisition: PC-MRI with three-directional velocity information is collected. **(2)** Preprocessing: data preprocessing corrects for errors due to noise, aliasing, and eddy current. **(3)** Segmentation: the centerline was segmented parallel traced along the target vessel. **(4)** Analysis: the plane is set at the narrowed segment of the lumen and segment of the lumen contour in magnitude-coded image manually, then we can get the cross section and sagittal section visually, as well as the axial and circumferential WSS curve and the quantified values. **(5)** Visualize: examples of WSS evaluated by 4D flow MRI at different time points; 4D flow MRI can display hemodynamic changes dynamically and visually in different cardiac cycles.

As displayed in Figure 3, 4D flow MRI data were imported into the CVI42 software (Version 5.6.6, Circle Cardiovascular Imaging, Calgary, Canada) for preprocessing and parameter calculation; the analysis steps included the following: (1) preprocessing: the preprocessing step consisted of automatic offset correction, signal aliasing correction, correction of flow direction, and dynamic previewing of images in all directions to identify and exclude images with poor quality. (2) Segmentation: the target vessel was segmented parallel to the centerline, which was traced along the vessel. (3) Calculation: the analysis plane was placed perpendicular to the centerline at the narrowest part of the carotid artery, and velocity (maximum and mean), axial WSS (maximum and mean), and circumferential WSS (maximum and mean) were measured at 20 time points. WSS was calculated based on the method described by Markl (Stalder et al., 2008). The 3D WSS reflected the total WSS along the plane tangent to the local vessel surface, and was decomposed into axial and circumferential components. Axial and circumferential WSS represent the WSS along the blood flow direction and the vessel circumference. The stenosis rate was calculated using HRVW imaging data and the equation for stenosis rate = D_s_/D_d_ or D_s_/D_p_), in which D_s_ is the diameter of the narrowed segment of the lumen, D_d_ is the diameter of the distal lumen next to the narrowed segment, and D_p_ is the diameter of the proximal lumen next to the narrow segment. All statistical analyses were performed with SPSS (Version 19.0.0 IBM, Armonk, NY, United States). The stenosis rate and volume were compared using independent-sample *t*-tests. The WSS and velocity values displayed normal distributions according to a normality test. An independent-sample *t*-test was used to compare WSS (axial, circumferential and 3D) and velocity between low- and high-risk plaques over the entire cardiac cycle and at 20 different time points; *p* < 0.05 was considered statistically significant.

## Results

The demographics of the patients with high-risk plaques and low-risk plaques are shown in Table 1. There were no significant differences in stenosis or volume between the high-risk and low-risk groups. Among the 30 patients (20 male, 56.3 ± 9.5 years), 19 patients had hypertension, 6 patients had hyperlipidemia, 16 patients were active smokers, and 6 patients had diabetes. The mean heart rate was 75.0 ± 10.8 times/min.

**TABLE 1:** Patient demographic data.

Characteristics	N (%)	*P*
Patient characteristic*		
Age (years)^a^	56.3 ± 9.5	
Male	20 (66.7%)	
Smoke	16 (53.3%)	
Hypertension	19 (63.3%)	
Diabetes	6 (20.0%)	
Hyperlipidemia	6 (20.0%)	
Heart rate (times/min)^a^	75.0 ± 10.8	
**Plaque identification^†^**		
High-risk	24 (60.0%)	
Low-risk	16 (40.0%)	
Stenosis rate^a†^		0.359
High-risk	53.2 ± 7.9	
Low-risk	50.9 ± 7.4	
Volume in the stenosis lumen (ml)^a†^		0.965
High-risk	3.6 ± 2.0	
Low-risk	3.5 ± 1.3	

^a^Mean (± SD). *For the entire cohort of 30 patients. ^†^For the 40 carotid arteries with 40 plaques.

**FIGURE 4:**
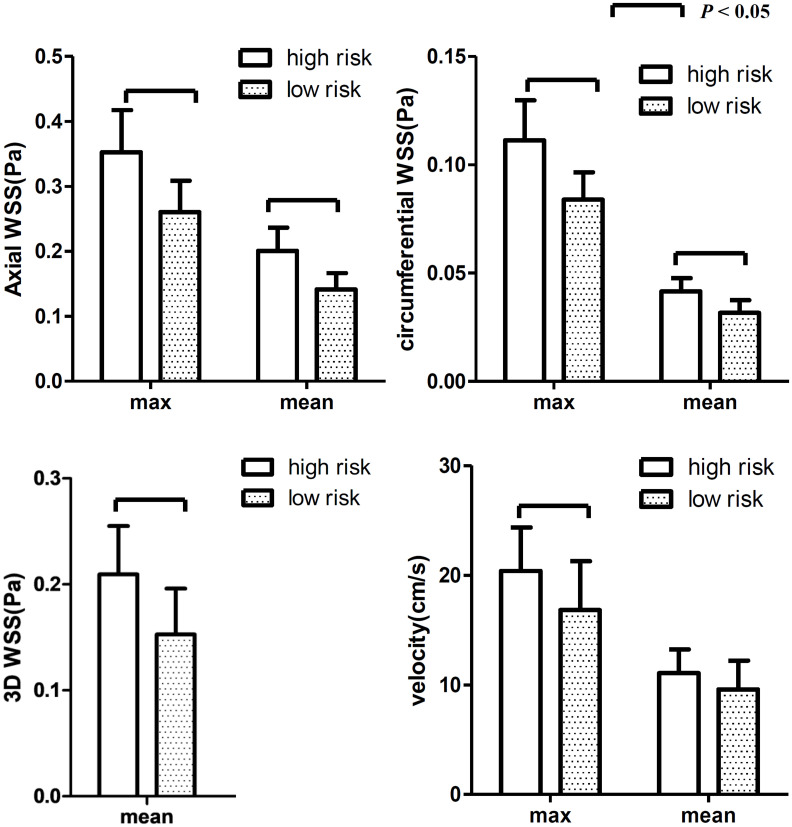
The differences in WSS (axial, circumferential, and 3D) and velocity at all 20 time points. The WSS values were higher in the high-risk group than those in the low-risk group. Both max and mean WSS values between the two groups were found significant, while no significant difference was found for mean velocity.

The maximum and mean values of axial, circumferential, and 3D WSS and velocity at all time points were all higher in the high-risk plaque group than those in the low-risk plaque group. Figure 4 shows that the differences were significant except for mean velocity (*p* = 0.061).

**FIGURE 5:**
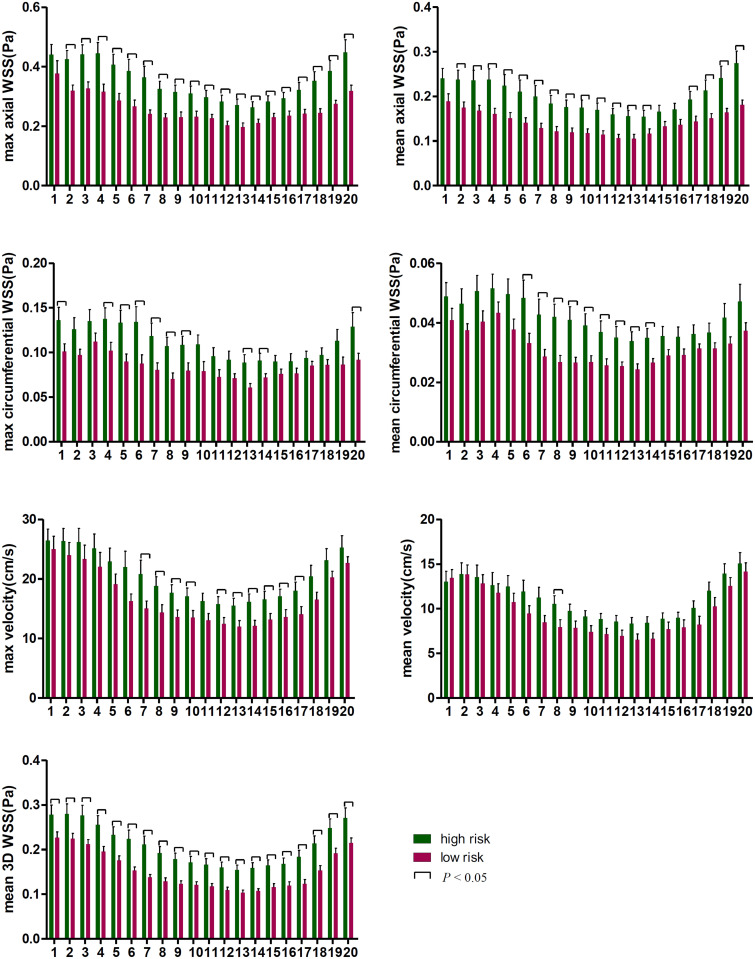
The WSS differences at every time point in the cardiac cycle. The first time point was the start of the rapid ejection period, with 1–5 time points and 16–20 time points representing systole and 6–15 time points representing diastole.

The differences at each time point were also analyzed separately. Figures 4, 5 show that the value of axial WSS was significantly higher than that of circumferential WSS, the WSS was 0.18 ± 0.08 Pa (mean ± SD) in the axial direction and 0.04 ± 0.02 Pa (mean ± SD) in the circumferential direction (*P* < 0.001), and the WSS value in the axial direction was more than four times that in the circumferential direction. The differences in circumferential WSS at half of the time points were non-significant, whereas non-significant maximum and mean axial WSS values were observed at only one time point and three time points, respectively. The difference in 3D WSS was significantly different between the two groups at all time points. The trend of 3D WSS more closely resembled that of axial WSS than that of circumferential WSS, but 3D WSS was more stable than axial WSS at all different time points.

The WSS and velocity differed between systole and diastole within a cardiac cycle. As displayed in Figure 5, WSS was higher in the high-risk plaque group than in the low-risk plaque group at each time point, but the difference was non-significant (*p* > 0.05) at a few time points, especially during systole. The number of non-significant differences in each WSS variable was as follows: 1 (systole) in maximum axial WSS, 2 (systole) and 1 (diastole) in mean axial WSS, 6 (systole) and 4 (diastole) in maximum circumferential WSS, and 10 (systole) and 1 (diastole) in mean circumferential WSS. Velocity was higher in the high-risk plaque group than in the low-risk plaque group, except for the first time point (systole) for mean velocity, and the differences in the maximum diastolic velocity were all significant (all *p* < 0.05).

Figure 3 (5.Visualize) displayed the cases of dynamic changes in 3D WSS at different time points. The WSS in the high-risk plaque group increased at stenosis, and the degree of changes varied at different time points. The WSS of low-risk plaques declined in the stenotic location.

## Discussion

WSS plays an important role in the development of atherosclerosis. We studied the difference in WSS in vessels with similar degrees of stenosis using 4D flow MRI. Our study demonstrated that high-risk plaques had higher WSS than low-risk plaques. The WSS changes varied at different points of the cardiac cycle, and diastolic WSS may have a greater impact on plaque stability than systolic WSS. The axial WSS values were significantly higher than the circumferential WSS values. Additionally, 3D WSS best reflected the difference between high-risk and low-risk plaques in that it had a smaller standard deviation than the other WSS parameters, and it showed a significant difference at every time point. 4D flow is a notable emerging technology to display changes in any hemodynamic parameters at different time points in the cardiac cycle visually and dynamically; it is an *in vivo* technology and provides multidirectional and multi-time-phase information. Its hemodynamic measures can be combined with anatomical information to help clinicians make more accurate judgments based on a patient’s clinical condition.

Vessel stenosis greater than 70% is independently associated with cerebrovascular events and is considered a high-risk feature. Studies have found that moderate stenosis is also associated with a high incidence of cerebrovascular events (Bodle et al., 2013), and stenosis is not an independent predictor in defining plaque risk (Shi et al., 2018; Shi et al., 2020). Therefore, it is of great importance to identify high-risk plaques in patients with moderate stenosis (Shi et al., 2021). Previous studies have mostly addressed HRVW imaging, whereas *in vivo* hemodynamic studies have been less extensively discussed. WSS is the force generated by blood flow and is directly perpendicular to the vessel wall and the plaque. The “axial” here intended as the direction aligned with the tangent to the vessel’s centerline and the main flow direction, and the “circumferential” intended as the direction was along the lumen circumference, orthogonal to the axial direction and centerline (Stalder et al., 2008; Morbiducci et al., 2015). Measurements at time points represent different times in the cardiac cycle; the magnitude of the force varies with the contraction of the heart. High WSS was related to induce specific changes in endothelial cell behavior, exacerbating inflammation and stimulating progression of the atherosclerotic lipid core in the vessel wall; it was a possible causative factor to promote the development of high-risk plaques (Eshtehardi et al., 2017). Understanding the detailed changes in plaque WSS can help clinicians apply more active treatments when a plaque shows high WSS, especially in diastolic and axial WSS.

Previous studies (Groen et al., 2007; Tuenter et al., 2016) found that ulcers formed exclusively at locations of high WSS, and a higher maximum WSS was significantly associated with the presence of IPH. Gijsen et al. (2011) found that plaque regions exposed to high WSS were subject to increasing strain over time, indicating that high WSS was likely to increase lipid deposition. These studies roughly corresponded to ours, although our study was more detailed in that we investigated axial, circumferential, and 3D WSS *in vivo* using 4D flow MRI. WSS can affect the function of vascular endothelial and smooth muscle cells, and high WSS induces specific changes in endothelial cell behavior, for example, by modifying gene expression, which may contribute to the onset and progression of atherosclerosis (Castier et al., 2005; White et al., 2011; Eshtehardi et al., 2017).

The ability to detect hemodynamic changes at different time points in the cardiac cycle is a feature of 4D flow MRI, and studies about these changes in the carotid artery are rare. A 4D flow MRI study of WSS changes in regurgitant semilunar valvular lesions by Rizk et al. (2019) found that in the presence of pulmonary and aortic regurgitation, WSS was elevated in comparison with controls and showed a diastolic peak. In contrast, Geiger et al. (2018) found that systolic WSS was not significantly altered in bicuspid aortic valve (BAV) patients. Our research also found that WSS values were increased throughout the entire cardiac cycle, but the difference in diastolic WSS was more significant than that in systolic WSS. Although previous work explored different diseases than our study did, these prior studies explained the role of diastole in the presence of disease; this role cannot be ignored and may be a considerable contributing factor to some cardiovascular diseases. The diastolic period lasts longer than the systolic period, consequently affecting the vessel wall longer; therefore, diastole may play a more important role in WSS.

Choi et al. (2015) explored the relationship between axial WSS and plaque, finding that upstream axial plaque stress increased with lesion severity and that axial plaque stress showed a negative correlation with lesion length. These results were complementary to ours. Furthermore, we further compared the contributions of axial WSS and circumferential WSS to plaque risk, and we found that axial WSS may be more meaningful in the assessment of plaque risk and in the selection of treatment strategies for patients with atherosclerotic plaque. Studies on other diseases, such as Marfan syndrome and BAV (Bissell et al., 2013; Wang et al., 2016; Guala et al., 2019), found that these patients exhibited reduced axial and circumferential WSS, but in different locations, circumferential WSS was reduced in the distal ascending aorta and in the proximal descending aorta in patients with Marfan syndrome, whereas axial WSS displayed more dilatation at the aortic root, and greater circumferential WSS was positively associated with dilatation in the ascending aorta in BAV patients. 3D WSS is the summation of the axial and circumferential WSS, Patients with high-risk plaques had greater WSS than those with low-risk plaques, but some differences in the axial and circumferential WSS were not statistically significant. When the differences in axial and circumferential WSS were combined for analysis, the difference in 3D WSS was significant at every time point. Further investigations will be needed in the future to address the specific reason, but it can be speculated that shear stress acting on the vessel wall axially rather than circumferentially plays a key role in high-risk plaques.

Some limitations in our study should be mentioned. First, our criteria for high-risk plaques required not only imaging results but also cerebrovascular events. A few patients had cerebrovascular events but no high-risk imaging signs, or vice versa, and were not included in the high-risk group. This may limit the applicability of our results in the clinic. However, the purpose of our study is to identify the hemodynamic differences between high- and low-risk plaques; thus, the criteria for including high-risk plaques are stricter than those that are used in clinic. We believe that the inclusion of unmatched data in future studies will enrich this area of research. Second, the geometries and multi-slice (measuring several planes along the vessel), multi-segment (showing the external or internal wall of the internal carotid artery, where the stenosis presented) models of the carotid arteries, which have been discussed in previous studies, were not investigated in this study, although they may provide more detailed and comprehensive information. Third, the amount of sample data is relatively small, and a larger dataset is need for further verification in the future.

## Conclusion

In conclusion, 4D flow MRI can display hemodynamic changes dynamically and visually across cardiac cycles. Patients with high-risk plaques were found to have higher WSS than those with low-risk plaques. The increases in axial WSS were greater than those in circumferential WSS. Diastolic WSS was more influential than systolic WSS in determining plaque outcomes. High WSS, especially diastolic and axial WSS, may be related to atherosclerotic plaque rupture and cause cerebrovascular events in patients with moderate carotid artery stenosis.

## Data Availability Statement

The original contributions presented in the study are included in the article/supplementary material, further inquiries can be directed to the corresponding author/s.

## Ethics Statement

The studies involving human participants were reviewed and approved by the Institutional Review Board of Tongji Hospital, Tongji Medical College, Huazhong University of Science and Technology, Wuhan, China. Written informed consent for participation was not required for this study in accordance with the national legislation and the institutional requirements.

## Author Contributions

GZ and WZ were responsible for the study concepts and design. GZ, SZ, YQ, XT, LL, WL, and WZ were responsible for literature research. GZ, JF, YZ, DW, and WZ were responsible for the clinical studies. GZ and SY were responsible for the statistical analysis. All the authors were guarantors of integrity of the entire study and responsible for the experimental studies and data analysis, manuscript preparation and editing, and final approval.

## Conflict of Interest

The authors declare that the research was conducted in the absence of any commercial or financial relationships that could be construed as a potential conflict of interest.

## Publisher’s Note

All claims expressed in this article are solely those of the authors and do not necessarily represent those of their affiliated organizations, or those of the publisher, the editors and the reviewers. Any product that may be evaluated in this article, or claim that may be made by its manufacturer, is not guaranteed or endorsed by the publisher.
